# Risk Factors for Inpatient Hospital Admission in Pediatric Burn Patients

**DOI:** 10.7759/cureus.602

**Published:** 2016-05-06

**Authors:** Alvin To, Yana Puckett

**Affiliations:** 1 Saint Louis University School of Medicine; 2 Surgery, Texas Tech University Health Sciences Center

**Keywords:** burns, patient admission, pediatrics, periodicity, seasons, risk factors, epidemiology, public health

## Abstract

**Purpose:**

Our objective was to determine the risk factors for inpatient admission of pediatric burn patients.

**Materials & methods:**

This cross-sectional study uses data from the Healthcare Cost and Utilization Project Kids' Inpatient Database (HCUP KID) for the years of 2003, 2006, 2009, and 2012 to estimate the risk factors for inpatient admission for pediatric patients who sustained a burn injury. Patients who sustained a burn between the ages of 1 and 18 years were included.

**Results:**

A total of 43,453 patients met inclusion criteria. Of those, 42.3% were Caucasian, 20.1% were African American, and 19.3% were Hispanic. Males comprised 63.5% of the studied population. The month of July was associated with a 31.8% increased chance (p=.011) of being admitted to hospital for a pediatric burn. It was found that patients being admitted had a 32.2% increased chance (p=.002) of a fluid and electrolyte abnormality and a 61.0% increased chance (p=.027) of drug abuse.

**Conclusions:**

Pediatric burn patients are more likely to be admitted to the hospital having a fluid and electrolyte abnormality, having a drug abuse status, and/or during the month of July.

## Introduction

In the United States, burn injuries affect a million children annually [1]. The cost of pediatric burn-associated hospitalizations and the total cost of burn-associated expenditure are estimated at around $211,772,700 and $2.1 billion, respectively [2-3]. Up to 91.3% of burn injuries are accidental; these cases may potentially be prevented by active strategies, such as educating at-risk populations on behavioral changes and environmental control, and passive strategies, such as installing safety devices [4]. Categorizing risk factors for burns may aid in devising burn prevention strategies.

Over 50% of pediatric burn injuries occur in children under the age of five with scald injuries being the major etiology [4]. Accidental burns are more likely to occur if the child is either in a dangerous environment or insufficiently supervised. As the safety of the environment and degree of supervision varies temporally, it follows that there may be a temporal pattern of burn injury presentation corresponding with the time of the day, day of the week, or month/season of the year. All-age studies around the world have presented discordant conclusions: some findings suggest increased prevalence of admission during summer [5-9], winter [10], both summer and winter [11-12], or both winter and spring [13-15]; some studies found no correlation with the seasons [16-18]. It is likely that population-specific factors, such as cultural norms, ethnicity, or local climate, may alter the behaviors of each study sample differently. Some of these behaviors may place individuals in environments that pose a greater risk for accidental burns, specific to certain months of the year. Within the United States, pediatric and all-age studies examining seasonal variation of burn risk have been limited in number and only represent a limited geographic range [5, 11, 19]; thus, there is a need to investigate admission patterns in the United States, which may guide prevention or burn treatment strategies.

In this study, we retrospectively examine a national pediatric database for burn injuries and inpatient admission. We assessed risk factors for admission including weekend status, month of admission, race, and payer status. Consent was obtained by all participants in this study.

## Materials and methods

Using data from the Healthcare Cost and Utilization Project Kids’ Inpatient Database (HCUP KID) compiled for the years of 2003, 2006, 2009, and 2012, we performed a retrospective analysis of all pediatric burn patients. Patient-related variables obtained from the database included age (years), sex, race (Caucasian, African American, Hispanic, Asian or Pacific Islander, Native American, and other), in-hospital death, fluid and electrolyte disorder, and history of drug abuse. Each record contained discharge diagnosis and procedure codes defined by the 15th International Classification of Diseases, Ninth Revision, Clinical Manifestations (ICD-9-CM). ICD-9 codes included 940.0-949.5.

Comorbid conditions were identified on the basis of both the ICD-9 diagnosis and procedure codes and the Clinical Classification Software diagnosis and procedure classifications. Statistical Package for the Social Sciences (SPSS) (IBM Corporation, New York, USA) was utilized for data analysis. Logistic regression was utilized to evaluate effect of month of admission, weekend status, gender, race, fluid and electrolyte abnormalities, drug abuse, age, and procedures required for all pediatric burn injuries on admission from 2003 to 2012. Estimations with P values less than .05 were considered statistically significant.

## Results

A total of 43,453 patients were analyzed. The average age of patients was 6.35 (SD=8.37), and 63.5% were male. Caucasians accounted for 42.3% of the population; African Americans, 20.1%; and Hispanics, 19.3%. Medicaid was the most common form of payment (51.3%), followed by private insurance (34.4%), and self-pay (6.9%). A total of 203 patients with burns did not survive, and the average number of procedures on admission was 2.08 (SD=3.1) (Table 1).

**Table 1:** Demographics of National Weighted Pediatric Burn Patients Across United States From 2003-2012 (N=43, 453)

Variable		Mean (SD)	Percent (N)
Age		6.35 (8.37)	
Male gender			63.5% (27,310)
Mortality			0.5% (203)
Number of procedures on admission		2.08 (3.1)	
Race			
	Caucasian		42.3% (16,388)
	African American		20.1% (7771)
	Hispanic		19.3% (7475)
	Asian or Pacific Islander		2.5% (970)
	Native American		0.9% (362)
	Other		7.5% (2902)
Primary expected payer			
	Medicare		0.2% (79)
	Medicaid		51.3% (22,272)
	Private Insurance including HMO		34.4% (14,930)
	Self-pay		6.9% (2,991)
	No charge		0.2% (69)
	Other (title v, worker’s compensation, CHAMPUS/CHAMPVA, other government)		7.1% (3,065)
Obesity			0.6% (255)
Fluid and electrolyte disorder			6.1% (2662)

The effects of month of admission, weekend status, gender, race, fluid and electrolyte abnormalities, drug abuse, age, and procedures required for all pediatric burn injuries on admission from 2003 to 2012 were evaluated by logistic regression. The results revealed that a pediatric burn patient was 31.8% more likely to be admitted to hospital in the month of July compared to other months (p=.011). The months of January and April were associated with decreased chances of admission by 44% and 13%, respectively.

African Americans and Hispanics pediatric burn patients were associated with decreased odds of admission by 15% and 30%, respectively. Gender was not found to be statistically significant for odds of inpatient admission in pediatric burn patients. Patients had a two percent increased chance of being admitted to the hospital if a procedure was required (p=.001).

Children who were younger than five years old were less likely to be admitted by 25% (p<.0001) compared to children older than five years of age. However, ages between six to ten and 11-15 were not statistically significant for either an over- or under-representation of inpatient admission.

Fluid and electrolyte abnormalities and drug abuse were factors associated with need for admission to hospital by 32.3% and 61%, respectively. Private insurance payment type was associated with a decreased chance for inpatient admission by 30% (p<.0001) (Table 2).

**Table 2:** Logistic Regression Models Effect of Month of Admission, Weekend, Gender, Race, Fluid and Electrolyte Abnormalities, Drug Abuse, and Age, Procedures Required for Pediatric Burn Injuries on Admission from 2003-2012 (N=28, 905).

Variable		Admission Odds Ratio (95% C.I.)	p
Age (years)			
	0-5	.751 (.678-.833)	.000
	6-10	1.098 (.963-1.253)	.164
	11-15	1.123 (.981-1.285)	.164
Sex (female)		.879 (.396-1.952)	.751
Procedures required on admission		1.020 (1.008-1.033)	.001
Mortality		2.926 (1.323-6.469)	.008
Weekend admission		2.386 (2.131-2.671)	.000
Month of admission			
	January	.563 (.366-.866)	.009
	April	.868 (.683-1.104)	.003
	July	1.318 (1.065-1.632)	.011
Race			
	Caucasian	1.190 (.964-1.469)	.105
	African American	.850 (.720-1.003)	.054
	Hispanic	.700 (.582-.842)	.000
Payment type			
	Medicare	.000 (.000-.000)	.999
	Medicaid	1.252 (.616-2.543)	.534
	Private insurance including HMO	.696 (.609-.794)	.000
	Self-Pay	.986 (.862-1.127)	.831
	No Charge	.774 (.644-.924)	.006
	Other (title v, worker’s compensation, CHAMPUS/CHAMPVA, other government)	.472 (.138-1.616)	.232
Obesity		1.407 (.792-2.500)	.244
Fluid and electrolyte abnormality		1.322 (1.105-1.581)	.002
Drug abuse		1.610 (1.056-2.454)	.027

## Discussion

In this study, pediatric-burn associated hospitalizations were examined from a national sample of cases using HCUP KID data. Current findings on the seasonal distribution of pediatric burn admissions have been limited in sample size and geographic scope, and as a result, present discordant conclusions. Our study is based on a national dataset and suggests a 31.8% increased likelihood for a burn-associated pediatric patient to be admitted during July. Furthermore, to our knowledge, this study is the first to examine a national burn injury population and establishes a correlation between odds of admission with fluid and electrolyte abnormalities and/or drug abuse status.

In this analysis, patients identifying as African American and Hispanic comprise 20.1% and 19.3% of the pediatric burn cases, respectively. In contrast, census data indicates that African Americans and Hispanics comprise 12.6% and 16.3% of the general population, respectively [20]. These demographic groups are overrepresented in pediatric burn cases within the United States—a finding also in line with previous research [4].

Interestingly, our analysis showed that despite over-representing the pediatric burn population, African Americans and Hispanics had 15% and 30% decreased odds, respectively, of being admitted compared to Caucasians. The decreased odds of admissions may be explained by an increase in non-urgent cases in these demographics. It has been suggested that the Emergency Medical Treatment and Active Labor Act (EMTALA) has increased the incidence of emergency department usage by uninsured patients who have non-urgent medical injuries [21-22]. Nationally 11.8% and 19.9% of African Americans and Hispanics are uninsured, respectively; in comparison, 10.1% of Caucasians are uninsured [23]. Increased visits by patients with non-urgent needs could elevate the number of reported burn injuries. These minor burn injuries may be treated in the emergency department without need for hospital admission, thus lowering the apparent odds of admission.

Previous studies investigating temporal variations of burn inpatient admissions in the United States have had limited sample sizes representing a few states [5, 11, 19]; these studies’ conclusions have not been congruent and can only be applied to the state where each study was conducted. Our analysis represents pediatric burn patients nationally and shows a 31.8% increased likelihood for patients to be admitted during the month of July. Some smaller studies have demonstrated higher rates of hospital admissions during winter months, correlating with increased scald injuries possibly caused by consumption of hot beverages and use of electric heaters [11]. Our data, however, does not suggest a national increase of burn injury admissions during winter months.

Our observation of an increased risk of admission during July may represent risks that are more general to the national population. The Fourth of July holiday, celebrated with fireworks, may pose a significant burn risk. Annually, around 10,000 firework-related injuries occur in the United States [24]. Furthermore, 60% of firework injuries involve pediatric populations predominantly affecting 10-14 year old males [25]. A study found that pediatric firework injuries are 5.94 times more likely to occur within five days around Fourth of July compared to other holidays [26]. It should be noted, however, that 91.6% of children who receive emergency treatment for firework-related injuries are not admitted. Therefore, Fourth of July related firework burns might not sufficiently account for the increase in pediatric burn admissions in July. Increases in temperature around summer may be an additional contributor. Small sample studies have documented that infants and preschoolers are at risk of plantar burns caused by sun-heated surfaces at home and in public play areas [27-28].

Pediatric burn patients who presented with a history of drug abuse (amphetamines, barbiturates, opioids, and/or cannabinoids) were more likely to be admitted by 61% (p=.027). Harmful behaviors associated with substance abuse increases the risk of adverse burn outcomes [19, 29]. Up to 60.6% of incidents reported in these studies involved alcohol consumption; these patients had increased surface area of burn coverage, greater number of medical procedures, and lengthened hospitalizations. Within our study, burn patients presenting with a history of drug abuse may have been admitted to treat not only their burns, but also their substance abuse, which may explain the increased inpatient admission rate within this demographic.

Some limitations of this study need to be recognized. The study population represents individuals seeking emergency department care, including those who were subsequently admitted. Outpatient burn victim data is not recorded in HCUP KID. Minor burn cases may be significant, although underrepresented, in this study due to the possibility of outpatient self-medication. Therefore, the correlations observed in our study may be more applicable to moderate or severe burn cases presenting in the Emergency Department.

## Conclusions

This study reports a correlation between national pediatric burn admissions and a history of drug abuse and/or fluid and electrolyte abnormalities within the United States. Nationally, the risk for children to be admitted for burns is greatest during the month of July. These findings may assist the development of future strategies aimed at reducing pediatric burns.
